# Road Extraction from Unmanned Aerial Vehicle Remote Sensing Images Based on Improved Neural Networks

**DOI:** 10.3390/s19194115

**Published:** 2019-09-23

**Authors:** Yuxia Li, Bo Peng, Lei He, Kunlong Fan, Zhenxu Li, Ling Tong

**Affiliations:** 1 School of Automation Engineering, University of Electronic Science and Technology of China, Chengdu 611731, China; liyuxia@uestc.edu.cn (Y.L.); pengbo1@std.uestc.edu.cn (B.P.); fankunlong@std.uestc.edu.cn (K.F.); lizhenxu@std.uestc.edu.cn (Z.L.); tongling@uestc.edu.cn (L.T.); 2 School of Software Engineering, Chengdu University of Information Technology, Chengdu 610225, China

**Keywords:** road, UAV sensors, image processing, convolutional neural net

## Abstract

Roads are vital components of infrastructure, the extraction of which has become a topic of significant interest in the field of remote sensing. Because deep learning has been a popular method in image processing and information extraction, researchers have paid more attention to extracting road using neural networks. This article proposes the improvement of neural networks to extract roads from Unmanned Aerial Vehicle (UAV) remote sensing images. D-Linknet was first considered for its high performance; however, the huge scale of the net reduced computational efficiency. With a focus on the low computational efficiency problem of the popular D-LinkNet, this article made some improvements: (1) Replace the initial block with a stem block. (2) Rebuild the entire network based on ResNet units with a new structure, allowing for the construction of an improved neural network D-Linknetplus. (3) Add a 1 × 1 convolution layer before DBlock to reduce the input feature maps, reducing parameters and improving computational efficiency. Add another 1 × 1 convolution layer after DBlock to recover the required number of output channels. Accordingly, another improved neural network B-D-LinknetPlus was built. Comparisons were performed between the neural nets, and the verification were made with the Massachusetts Roads Dataset. The results show improved neural networks are helpful in reducing the network size and developing the precision needed for road extraction.

## 1. Introduction

Roads are vital components of infrastructure; they are essential to urban construction, transportation, military, and economic development. How road information can be extracted is a topic of significant research interest currently. Recently, Unmanned Aerial Vehicle (UAV) exploration has seen rapid development in remote sensing fields, advantageous for its high flexibility, targeting capability, and low cost [1].

Recently, deep learning has been widely used in the field of information extraction with remote sensing images. Liu et al. [2] used a full-convolution neural network to extract the initial road area, then filtered the interference pattern and connected the road fracture area according to the road length–width ratio, morphological operation, and Douglas-Peucker (DP) algorithm. Li et al. [3] proposed a Y-Net network including a two-arm feature extraction model and one feature fusion model. Liu et al. [4] introduced feature learning method based on deep learning to extract road robust features automatically, and proposed a method of extracting road centerline based on multi-scale Gabor filter and multi-direction, non-maximum suppression. Xu et al. [5] constructed a new road extraction method based on DenseNet through the introduction of local and global attention units, which can effectively extract road network from remote sensing images with local and global information. Zhang et al. [6] proposed a new network for road extraction from remote sensing images, which combines ResNet and U-Net. Wang et al. [7] proposed a road extraction method based on deep neural network (DNN) and finite state machine (FSM). Sun et al. [8] proposed a new road extraction method based on superimposed U-Net and multi-output network model, and used mixed loss function to solve the problem of sample imbalance in multi-objective classification. Kestur et al. [9] proposed U-FCN (U-type Fully Convolutional Network) model to realize road extraction from UAV low-altitude remote sensing images, which consists of a set of convolution stacks and corresponding mirror deconvolution stacks. Sameen et al. [10] proposed a high resolution, orthophoto road segmentation framework based on deep learning. In this method, a deep convolution self-encoder is used to extract the road from orthophoto image. Costea et al. [11] proposed three successive steps to extract the road in remote sensing images: (1) Extract the initial road results by combining multiple U-Net networks; (2) Use a Convolutional Neural Network(CNN) to recover the details of the output results in the first network; and (3) Connect the road breakage area by reasoning to improve the road extraction accuracy. Filin et al. [12] proposed a method combining deep learning and image post-processing to extract road information from remote sensing images. The road extraction results were improved through four steps: vectorization of road results, detection of road width, extension of interrupted roads, and removal of non-road areas. Buslaev et al. [13] constructed a full convolution network similar to U-Net structure by using ResNet_34 pre-trained in ImageNet. 

D-LinkNet [14] was introduced because it won the CVPR DeepGlobe 2018 Road Extraction Challenge because of its highest Intersection over Union (IoU) scores on the validation set and the test set. D-LinkNet could have higher IoU scores if it used ResNet, whose encoder part has a deeper layer than ResNet_34 [15]; however, the scale of model would be extremely large because of the design of its center part called DBlock [16]. To address this problem, the article proposed improved neural networks to extract road information based on D-Linknet: (1) Replace the initial block with stem block; (2) Rebuild the entire network based on ResNet units with a new structure so that a new neural network, D-Linknetplus, can be built; and (3) Add a 1 × 1 convolution as a layer before DBlock to reduce the input feature-maps, to reduce the parameters, and to improve computational efficiency. Meanwhile, add another 1 × 1 convolution layer after DBlock to recover the required number of output channels. Accordingly, an improved neural network B-D-LinknetPlus was built. The accuracy of road extraction results obtained by the modified model were trained using our own training dataset after data augmentation [17,18]. 

## 2. Materials and Methods

The UAV remote sensing image is the first processing material required for the research; however, the data obtained from the camera sensor has several disadvantages. Accordingly, we first had to take some transportation before labeling and training. Further, the convolution neural net was introduced for processing and extracting road information from UAV images.

### 2.1. Materials

#### 2.1.1. Image Data Source

**Figure 1:**
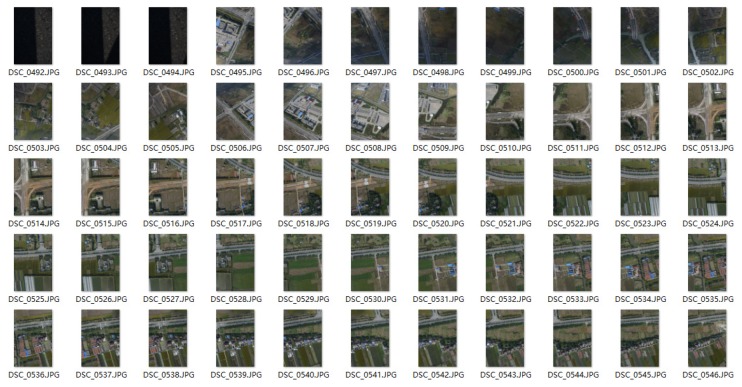
Unmanned Aerial Vehicle (UAV) Remote Sensing Images.

**Figure 2:**
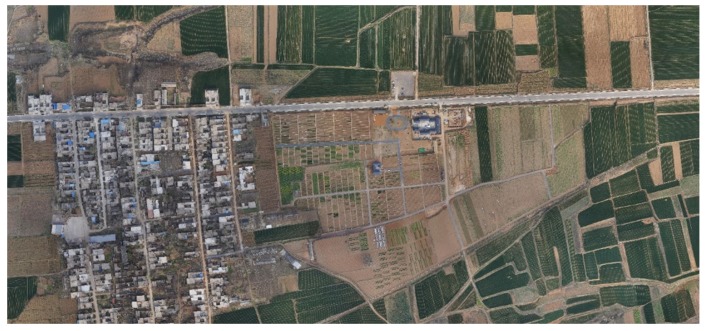
Mosaic Unmanned Aerial Vehicle images (Luoyang Region).

As seen in Figure 1, the experimental images from optical sensors were taken in different areas and at different times. The selected areas in the article are from the Luoyang region in Henan Province, as seen in Figure 2. The acquisition time of the UAV image in the Luoyang area was on 18 March 2018, and the original single image size is at the centimeter level. 

#### 2.1.2. Label Data Making

Because of the large number of parameters in the convolution neural network, if the model is directly applied to actual road detection after randomly initializing these parameters, the effect cannot be guaranteed. Therefore, before applying the convolution neural network to the actual situation, the network parameters first had to be trained. To evaluate the effect of network training, the first step was to manually train and test the samples.

**Figure 3:**
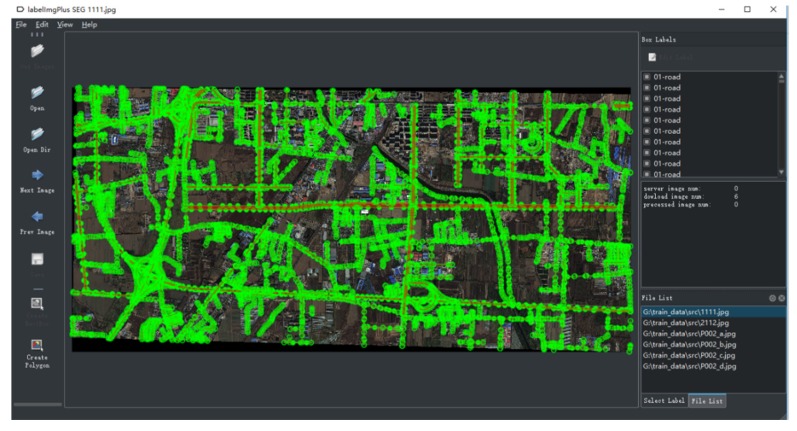
Initial Software Interface and Annotation Results.

This paper used label Img Plus-master, an open source sample annotation tool written in Python on GitHub, to label the images. The software combines Qt to create an accessible human–computer interaction interface. Because only the road information needs to be extracted for research, the road areas should be labeled and expressed in red on the software interface. The initial software interface and annotation results are shown in Figure 3. 

**Figure 4:**
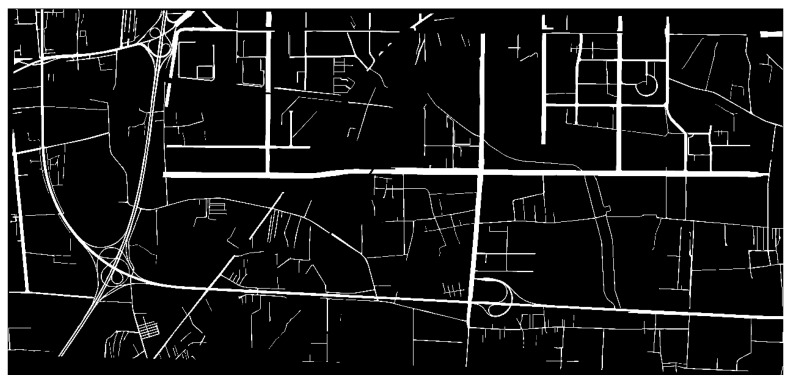
Label Result Image.

The road category label was set as 1, and the other areas labels were set as 0. To display the data conveniently, the pixel value of labeled area was set to 255. The labeled image is shown in Figure 4.

#### 2.1.3. Training Data Augmentation

The large number of samples required in a convolutional network greatly restricts its wide application. In this article, manual labeling of sample data was far from meeting the requirements of network training for the number of samples. However, prior research [17] fully demonstrated the effectiveness of data augmentation in network model training. Therefore, network training samples through data augmentation were added according to the actual scene. 

(1) Hue, Saturation, Value (HSV) Contrast Transform

If an image of the same area is taken at a different time, the information carried by the image differs because of changes in weather. An effective method is to transform the original image of training data from RGB color space into hue, saturation, and value (HSV) color space; subsequently, the details of the hue, saturation, and value for these images were changed to obtain the corresponding image in HSV color space. Further, the image was restored to RGB color space to obtain an additional training image. The change value of hue and saturation should not be too large; otherwise, the image restored to RGB color space will be seriously distorted. Reference [14] suggested that, in HSV color space, the change value of H channel, S channel, and V channel are set below 15%, 15%, and 30%, respectively. 

(2) Spatial Geometric Transformation

**Figure 5:**
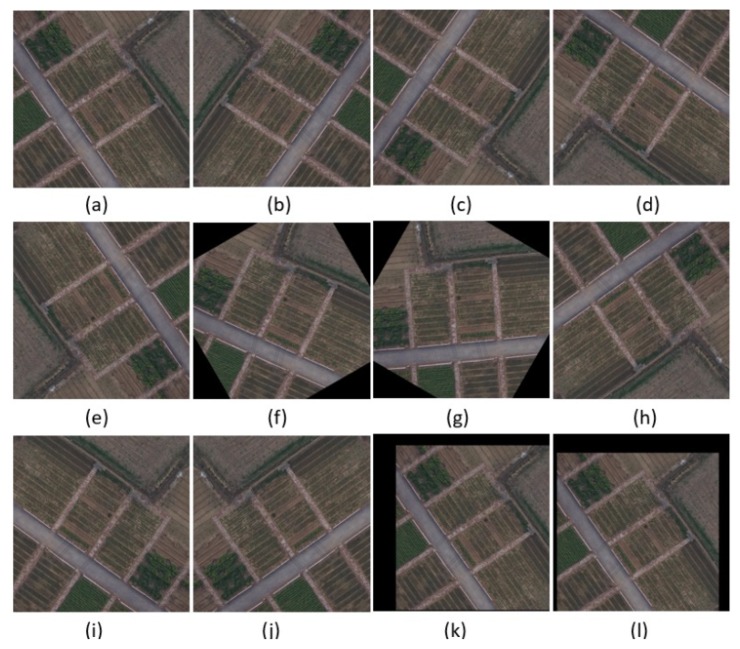
The results of spatial geometric transformation of the label (**a**) original label; (**b**) vertical flip; (**c**) horizontal flip; (**d**) main diagonal rotation; (**e**) sub diagonal transpose; (**f**) 30 degree clockwise rotation; (**g**) 60 degree clockwise rotation; (**h**) 90 degree clockwise rotation; (**i**) 180 degree clockwise rotation; (**j**) 270 degree clockwise rotation; (**k**,**l**) random shift and scaling (approximately −10–10%).

Label quality significantly impacts the deep learning model. When the label space or quantity is insufficient, it will seriously affect the training result or lead to the insufficient generalization degree of the trained model, which will reduce the recognition rate and accuracy rate. To obtain diverse label data, spatial geometric transformation including rotation, flip, zoom and affine transformation is necessary. Partial examples of transformation results are shown in Figure 5. The data augmentation was processed in an ambitious way according to References [14,15,17]. The road direction of original label is from west north to east south, as seen in Figure 5. After image geometric transformation, the road direction and its surrounding environment were changed greatly. For the training of convolutional neural network, more information about the image features can be acquired.

After HSV contrast transformation and spatial geometric transformation of the original image, the training label data significant increase in quantity, which is helpful for eliminating overfitting on the train set during convolution neural network training.

(3) Image Clipping

If large-sized training images are used directly for training, the demands on computer performance are higher and the training phase will be longer. In this experiment, the selected computer contained one RTX 2080 Ti graphics card and 32 GB running memory. Further, each training image was cut into a suitable size for this article. To guarantee that the net could learn the global characteristics, the overlap rate of adjacent images was set to 100 pixels. 

After some of the above steps, 47,152 pairs of training data and 1012 test data without data augmentation were obtained. To avoid the model being over-adapted to the image characteristics of a certain region in the training phase, the training data of all regions were arranged out of order and stored in a folder. The original image and the corresponding label image were stored in different folders. TensorFlow provides an efficient way to read training data; accordingly, the training image was generated into its standard format TF-Record. All training images were written into a single large file.

### 2.2. Methods

With the popularization of deep learning, designing a network structure has become simpler. However, a convolution neural network is stacked by common components such as convolution layer, pooling layer, and activation layer. Different network structures formed by different stacking orders as well as the number of these components have different effects on the accuracy of the results. Therefore, in a convolution network, the accuracy of the final result will be different. In practical applications, the problem is how to design a good network structure.

#### 2.2.1. Stem Block

**Figure 6:**
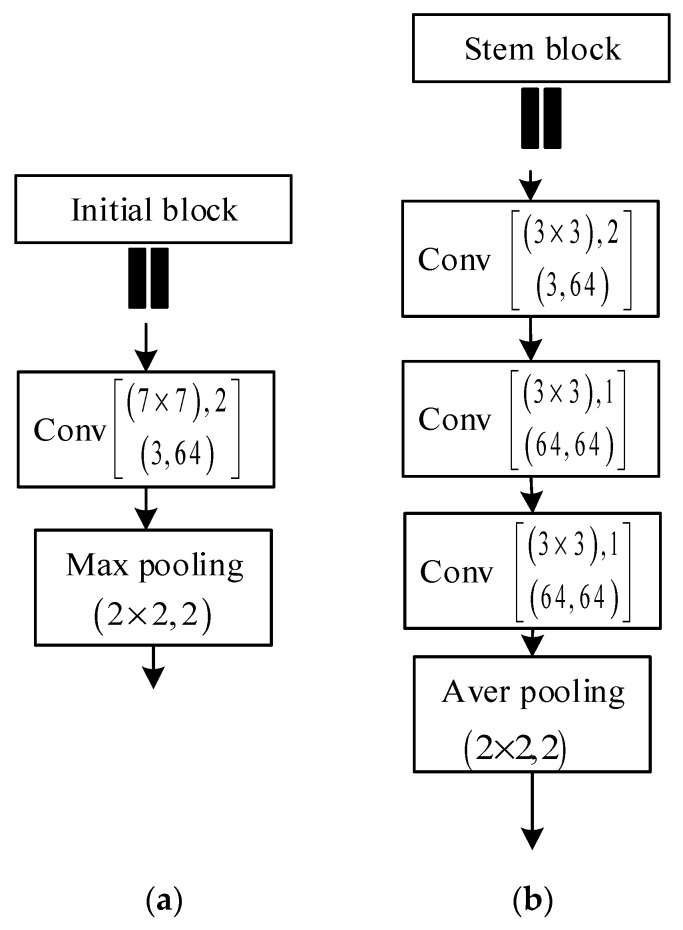
The contrast of initial block and stem block: (**a**) initial block and (**b**) stem block.

For D-LinkNet, there is an initial block [15] (7×7 convolution layer, stride = 2 followed by a 3×3 max pooling, stride = 2). However, reference [19,20,21,22] presented that two iterations of consecutive down-sampling will lose feature information, making it difficult to recover detailed information in the decoding stage. Inspired by Inception v3 [20] and Inception v4 [23], reference [24] replaced the initial block with stem block. The stem block consists of three 3×3 convolution layers and one 2×2 mean pooling layer. The stride of the first convolution layer is 2 and the others are 1. The output channels for all three convolution layers are 64. The experiment result in [19] demonstrated that the stem block will successfully detect objects, especially small objects. The contrast of initial block and stem block is shown in Figure 6.

#### 2.2.2. D-LinkNetPlus Building

When training a network, to obtain better precision, we constantly build a deeper network by stacking more layers. In an extreme case, the adding layers did not learn any useful features in terms of accuracy, but only duplicate the characteristics of the shallower layers onto the deeper layers, that is, the characteristics of the new layers identically map the shallow features. In this case, the performance of deep network is at least the same as that of the shallow network, and degradation should not occur. On the basis of this idea, He [15,16] proposed a new structure network, ResNet (Residual Neural Network). ResNet module can be expressed by the following Formulas (1) and (2):

(1) $$y_{l}=h\left(x_{l}\right)+F\left(x_{l},w_{l}\right)$$

(2) $$x_{l+1}=f\left(y_{l}\right)$$

where $x_{l}$ and $x_{l+1}$ indicate the input and the output of the lth residual unit. f indicates activation function while f indicates identity mapping, i.e., $x_{l+1}=y_{l}$ The formulas above can be shown as:

(3) $$x_{l+1}=x_{l}+F\left(x_{l},W_{l}\right)$$

So,

(4) $$x_{l+2}=x_{l+1}+F\left(x_{l+1},W_{l+1}\right)=x_{l}+F\left(x_{l},W_{l}\right)+F\left(x_{l+1},W_{l+1}\right)$$

The output of $L^{th}$ layer could be expressed as:

(5) $$x_{L}=x_{l}+\overset{L-1}{\underset{i=l}{\sum }}F\left(x_{i},W_{i}\right)$$

The feature of arbitrary level $x_{L}$ could be expressed by the previous $x_{l}$ and the residual structure $\sum_{i=l}^{L-1}F\left(x_{i},W_{i}\right)$. Therefore, even if the network is deep, the shallow features can be transmitted to the deep layers through identity mapping. If l=0 the formula could be shown as:

(6) $$x_{L}=x_{0}+\overset{L-1}{\underset{i=0}{\sum }}F\left(x_{i},W_{i}\right)$$

We find that the feature of the arbitrary layer could be obtained by adding input parameters $x_{0}$ and residual structure $\sum_{0}^{L-1}F\left(x_{i},W_{i}\right)$. So, the input could be jointed with output directly. Further, the gradient of any layer can be determined by the chain rule:

(7) $$\frac{\partial_{loss}}{\partial_{x_{l}}}=\frac{\partial_{loss}}{\partial_{x_{L}}}\cdot \frac{\partial_{L}}{\partial_{x_{l}}}=\frac{\partial_{loss}}{\partial_{x_{L}}}\cdot \left(1+\frac{\partial }{\partial_{x_{l}}}\overset{L-1}{\underset{i=l}{\sum }}F\left(x_{i},W_{i}\right)\right)$$

**Figure 7:**
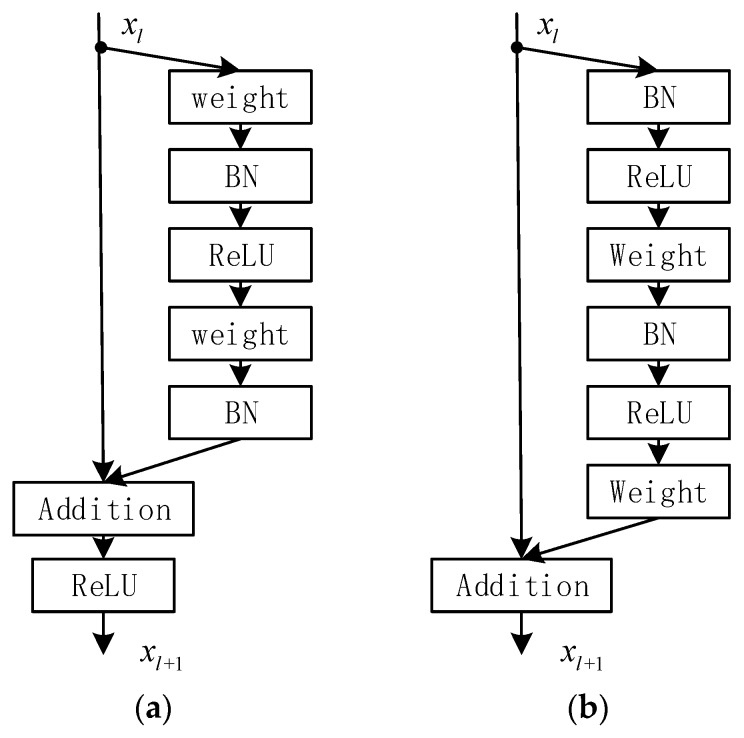
The structure of the different residual units: (**a**) Original residual unit and (**b**) New residual unit.

So, ResNet can address the problem of gradient dispersion very well even if the depth of the network is very deep. The original structure of residual unit is shown in Figure 7a. The output can be expressed by the following Formulas (8) and (9):

(8) $$y_{l}=x_{l}+F\left(x_{l},W_{l}\right)$$

(9) $$x_{l+1}=f\left(y_{l}\right)$$

where f indicates the activation function. The new structure of residual unit, ResNet variant, which was introduced in [16] is shown in Figure 7b. The output can be expressed by the following Formula (10):

(10) $$x_{l+1}=x_{l}+F\left(x_{l},W_{l}\right)$$

In contrast to the original residual unit, the new residual unit is essential for making information propagation smooth and obtain a higher accuracy, as seen in Figure 7.

**Figure 8:**
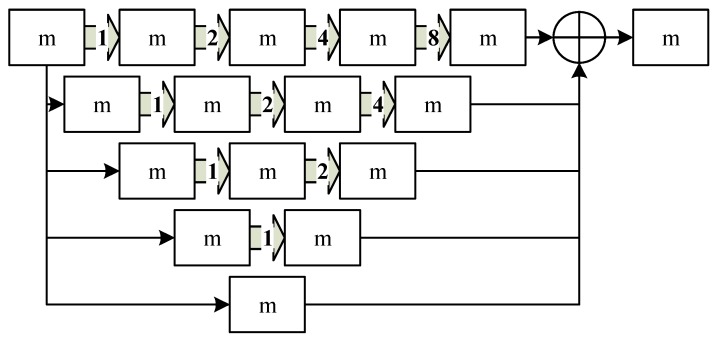
The structure of DBlock.

D-LinkNet uses several dilated convolution layers with central skip connections to increase the receptive field. It contains dilated convolution both in cascade mode and parallel mode. Each dilated convolution has a different dilation rate so the receptive field of each path is different; it can also combine features from different scales. D-LinkNet takes advantage of multi-scale context aggregation so that it has a great performance for road extraction. We called this center part as DBlock in this paper and its structure is shown in Figure 8. 

**Figure 9:**
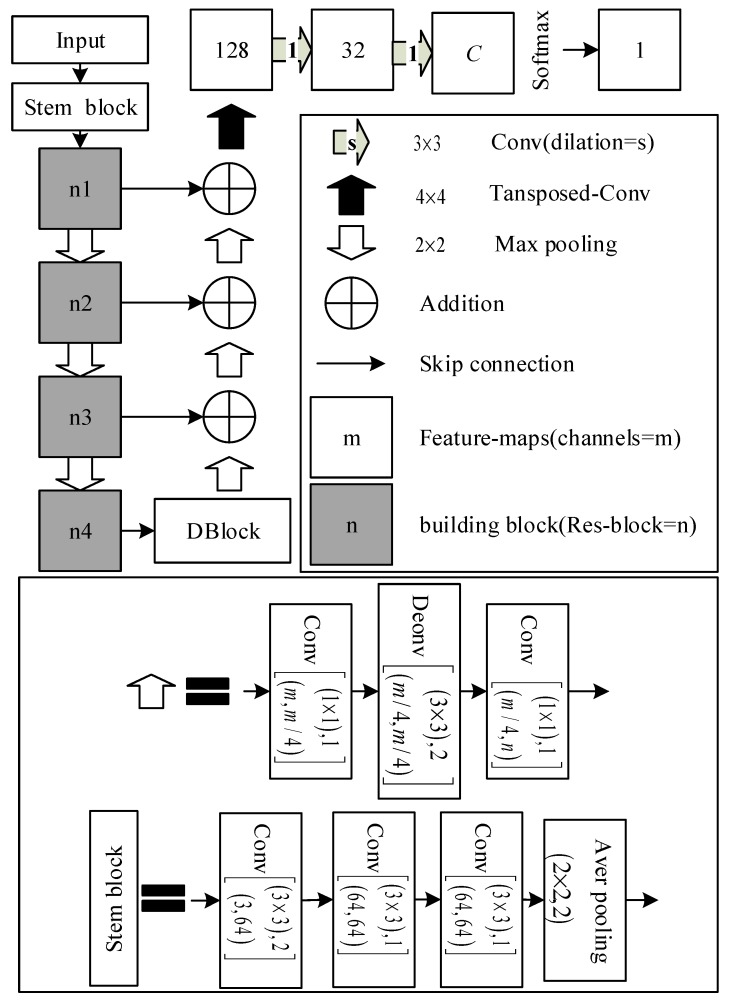
D-LinkNetPlus structure chart.

In conclusion, we rebuilt the D-LinkNet based on stem block and ResNet variant. The network called D-LinkNetPlus and its structure is shown in Figure 9. *C* indicates the classification category and the structure of Res-block is shown in Figure 7b. 

#### 2.2.3. DBlock Structure Optimization

**Figure 10:**
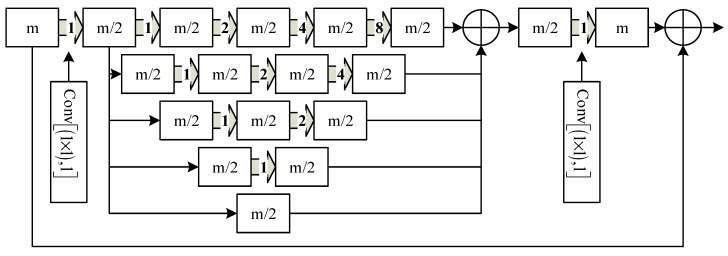
The structure of DBlockPlus.

Because the network structure of D-LinkNet came from the ResNet framework, the channel number of feature-maps increases gradually with deeper depths. At the end of the encoding stage, the number of output channels of feature-map is highest. Next, the feature-maps go through the DBlock and the output is taken as the input of the decoding stage. The number of input and output channels is the same in every dilated convolution, leading to numerous parameters throughout the whole network. Even if the network performance is excellent, the network scale would limit the application. The 1×1 convolution, introduced as a bottleneck layer in [15], is usually applied to change the number of channels of feature-maps, which can not only effectively fuse features but also greatly reduce the size of network parameters. This article added a 1×1 convolution before DBlock, which reduces the number of channels of the input feature-maps by half. Further, another 1×1 convolution was added after DBlock to recover the required channels. The structure, known as Dblock Plus, combines two 1×1 convolution layers with DBlock and its structure is shown in Figure 10. We replaced the DBlock with DBlockPlus in D-LinkNetPlus to build a new network that we called B-D-LinkNetPlus.

#### 2.2.4. Net Parameters

In this paper, the network structure of D-LinkNetPlus is constructed based on the ResNet variant. Using 50 layers and 101 layers [15] within the ResNet variant as the encoding stage, the network was built. The network called D-LinkNetPlus_50 and D-LinkNetPlus_101, respectively. The network parameters are shown in Table 1. The parameters of the B-D-LinkNetPlus are the same as D-LinkNetPlus.

## 3. Results and Discussion

### 3.1. Implementation Details

A RTX 2080 Ti graphics card was used for all network training and testing in this article. We used TensorFlow as the deep learning framework to train and test all networks. All the networks applied the same loss function defined in Equation (11):

(11) $$Loss=L2_{loss}+\overset{N}{\underset{i=1}{\sum }}BEC_{loss}\left(P_{i},GT_{i}\right)-lambda*Jaccard_{loss}$$

where $L2_{loss}$ represents L2 regularization loss for all the parameters in the model. $\sum_{i=1}^{N}BEC_{loss}\left(P_{i},GT_{i}\right)$ indicates binary cross entropy loss with N training images. P is the prediction results, and GT is label image. $\sum_{i=1}^{N}\left|P_{i}-GT_{i}\right|$ represents the differences between prediction results and label images on pixel-level. lambda means important the item is for total loss. The article suggests lambda to be set to around 0.7. Further, Adam was chosen as the optimizer. Following [14], the learning rate was originally set at 0.001 and reduced by 10 times while observing the slow decrease of the training loss. The learning rate decayed 0.997 times for every four-epoch size. The batch size during training phase was fixed as four; epoch size equals 2000.

### 3.2. Result and Discussion

#### 3.2.1. D-LinkNetPlus Result and Discussion

**Figure 11:**
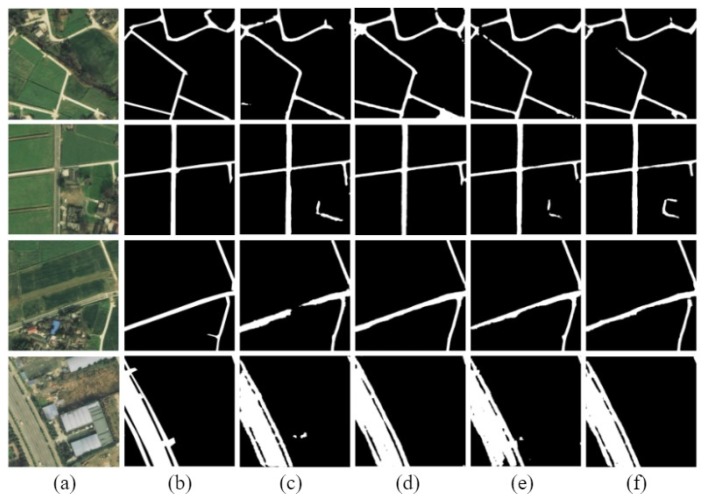
Comparison results of simple scene experiment. (**a**) Original image; (**b**) label image; (**c**) D-LinkNet_50; (**d**) D-LinkNet_101; (**e**) D-LinkNetPlus_50; and (**f**) D-LinkNetPlus_101.

**Figure 12:**
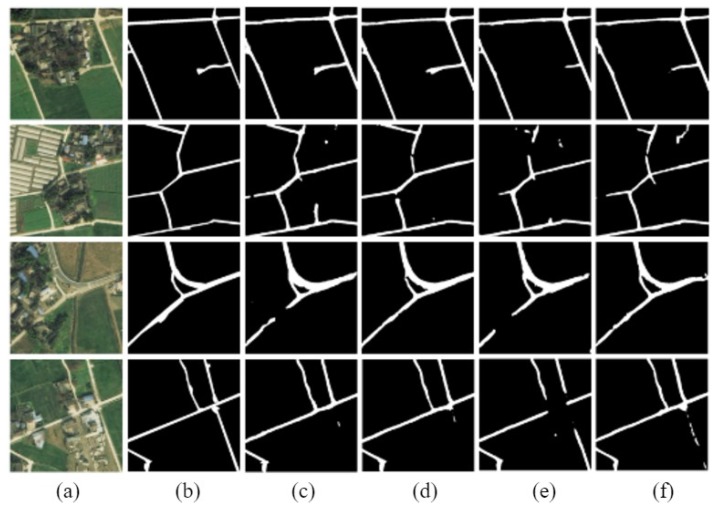
Comparison results of general scene experiments. (**a**) Input image; (**b**) label image; (**c**) D-LinkNet_50; (**d**) D-LinkNet_101; (**e**) D-LinkNetPlus_50; and (**f**) D-LinkNetPlus_101.

**Figure 13:**
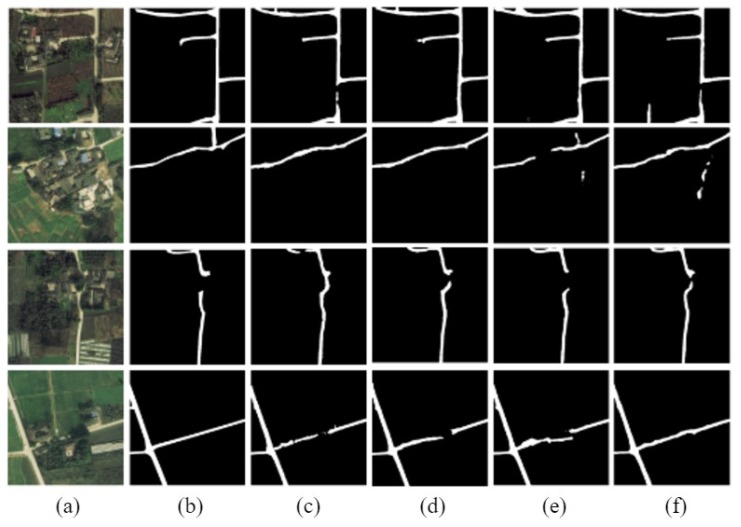
Comparison results of complex scene experiment. (**a**) Original image; (**b**) label image; (**c**) D-LinkNet_50; (**d**) D-LinkNet_101; (**e**) D-LinkNetPlus_50; and (**f**) D-LinkNetPlus_101.

The road extraction model of D-LinkNet, based on ResNet variant, is called D-LinkNetPlus. Similar to D-LinkNet, there are also 50 layers of D-LinkNetPlus_50 and 101 layers of D-LinkNetPlus_101 in the encoding stage. We divided the test images into three levels: simple scene, general scene, and complicated scene according to the complexity of image content scene. In the simple scene, the road areas are highlighted and there are small parts of the road areas that are covered by shadow. In a general scene, the road areas are around building groups; some parts of the road areas are covered by buildings, trees, and shadows. Other objects may have the same features as the road areas, such as highlighted greenhouses and highlighted buildings. These objects would affect the extraction results of the road areas. In a complicated scene, a long part of the road area is covered by other objects, such as trees or shadows, which make the road areas hard to extract. Partial experimental comparison results of three scene levels are shown in Figure 11, Figure 12 and Figure 13, respectively.

The new network with 101 layers in the encoding stage had better results than that with 50 layers in the simple scene experiments.

In the third row of the image, D-LinkNet_50 failed to detect a part of the road area blocked by the shadow of trees; however, the other three networks were able to connect the blocked area. The result boundary of the road area extracted by D-LinkNet_101 was smoother and closer to the manual label than that of D-LinkNetPlus_50 and D-LinkNetPlus_101. With regard to the highway in the fourth line, the extraction results from D-LinkNet_101 were better than those three network models not only broadly but also in terms of detail, which might be attributed to the fact that the stem block structure replaced the initial block structure.

The experimental results from D-LinkNet_101 of the general scene were better than those of the other three networks, but the overall effect was acceptable. The four networks can be detected for the peninsula ring area, where the two road materials intersect in the third line image. For the road in the lower left corner of the image in the fourth row, its shape and color are similar to the two roads in the upper right corner of the image. However, only D-LinkNetPlus could detect it, and the detection effect of D-LinkNetPlus_50 was better than that of D-LinkNetPlus_101, which may be due to the influence of the bare space on the left side of the road in the lower right corner.

As can be seen from Figure 13, the experimental results of D-LinkNet_101 show that the road area had better integrity without independent patches. From the third and fourth row of the image results, D-LinkNetPlus_101 is better than the other networks in connecting road area breakpoints blocked by buildings or trees. However, from the first and second row, many road areas are wrongly detected in the road extraction result of D-LinkNetPlus; many independent patches are left in the image, which will affect the calculation of the overall accuracy. 

The size of the network models, D-LinkNet, D-LinkNetPlus, and the calculated results of IoU accuracy, are shown in Table 2. The ResNet variant advances the nonlinear mapping element to a pixel by pixel addition. Theoretically, the two networks had the same model parameters; however, the size of D-LinkNetPlus is 100 megabytes less than that of D-LinkNet. According to the precision results calculated by IoU, the accuracy of D-LinkNetPlus_101 is higher than that of other three models, which is also consistent with previous analysis of the results of different scenes.

#### 3.2.2. B- D-LinkNetPlus Results and Discussion

**Figure 14:**
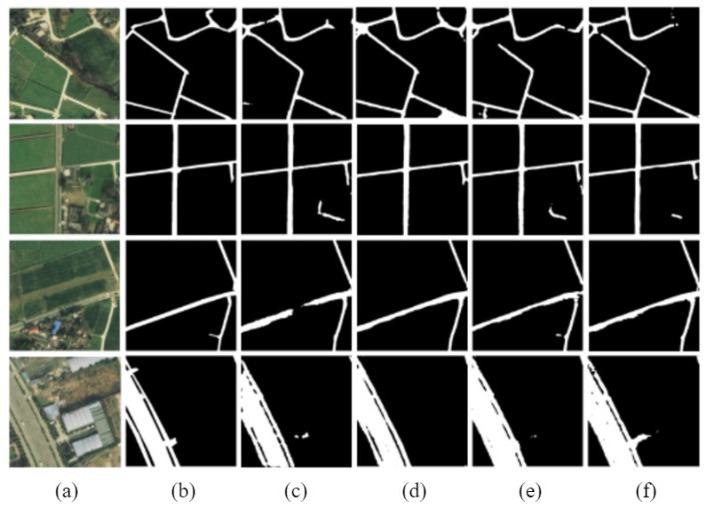
Comparison results of simple scene experiment. (**a**) Input image; (**b**) label image (**c**) D-LinkNet_50; (**d**) D-LinkNet_101; (**e**) B-D-LinkNetPlus_50; and (**f**) B-D-LinkNetPlus_101.

**Figure 15:**
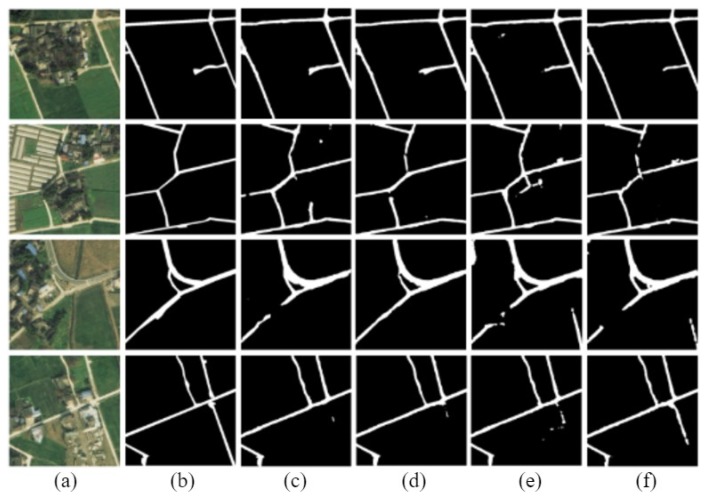
Comparison results of general scene experiments. (**a**) Input image; (**b**) label image; (**c**)D-LinkNet_50; (**d**) D-LinkNet_101; (**e**) B-D-LinkNetPlus_50; and (**f**) B-D-LinkNetPlus_101.

**Figure 16:**
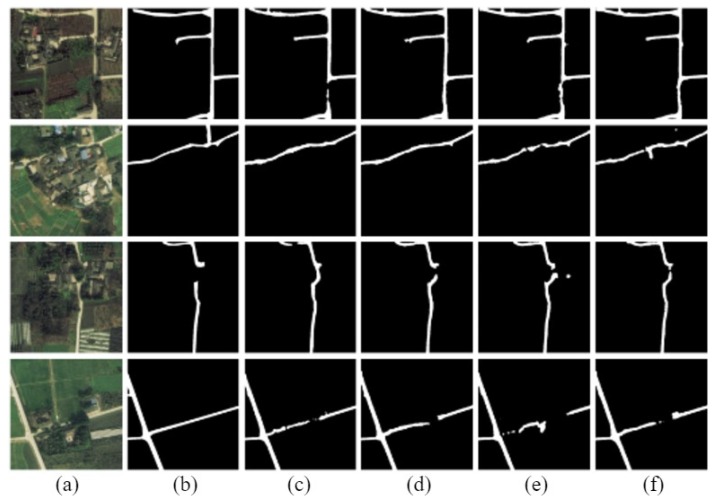
Comparison results of complex scene experiment. (**a**) input image; (**b**) label image; (**c**) D-LinkNet_50; (**d**) D-LinkNet_101; (**e**) B-D-LinkNetPlus_50; and (**f**) B-D-LinkNetPlus_101.

The D-LinkNetPlus with the bottleneck layer is called B-D-LinkNetPlus. Similar to D-LinkNetPlus, there are also 50 layers and 101 layers in the encoding stage, known as B-D-LinkNetPlus_50 and B-D-LinkNetPlus_101, respectively. A comparison of some experimental results of three scene levels is shown in Figure 14, Figure 15 and Figure 16, respectively.

As seen in Figure 14, with a focus on the network of D-LinkNet or B-D-LinkNetPlus, the image detection effect of layer 101 is better than that of layer 50 in the encoding stage. Further, fewer independent patches were left in the image and the road area overall had a higher integrity. From the details of road, D-LinkNet_101 is better than B-D-LinkNetPlus_101. But the high-road test showed that B-D-LinkNetPlus _101 was better than D-LinkNet_101, with neater edges.

As seen in Figure 15, D-LinkNet and B-D-LinkNetPlus have their own advantages in detection results for different scenes. In terms of connection road fracture, it can be seen from the third line picture that D-LinkNet has a better detection effect. However, for the field road area in the lower right corner of the third line image, this area can be judged as a road area or not a road area. Because this area does not belong to the main road, it was not marked as a road area when the label was made. Although B-D-LinkNetPlus detected this area, which cannot be considered as the false detection of the network, it will reduce the evaluation accuracy of the network.

As seen in Figure 16, whether using D-LinkNet or B-D-LinkNetPlus, the overall image road detection results are very close to the manual label images. However, in terms of broken road connection, D-LinkNet and B-D-LinkNetPlus are better than D-LinkNetPlus. With regard to the last line of images, the road area is covered by trees; accordingly, the characteristics of the road cannot be detected. However, they can be inferred according to the context concern, and the fractured area can be connected. 

The size of network models, D-LinkNet and B-D-LinkNetPlus, and the calculated results of IoU accuracy are shown in Table 3. In comparison with D-LinkNet, B-D-LinkNetPlus reduces the channel of input feature-maps of DBlock by half by adding 1×1 convolution before DBlock, greatly reducing model parameters and shortening training time.

#### 3.2.3. Validation on Public Dataset

To verify the effectiveness of the proposed method, we also tested our method with the Massachusetts Roads Dataset, which consists of 1108 train images and 14 validation images. The original image size was 1500 × 1500 pixels. The dataset was formulated as a binary segmentation problem, in which roads are labeled as foreground and other objects are labeled as background. We performed HSV Contrast Transform, Spatial Geometric Transformation, and image clipping on the original data set. Through training models and experiments, we obtained model size and IoU index. The size of the network models, D-LinkNet, D-LinkNetPlus, B-D-LinkNetPlus, and the calculated results of IoU index are shown in Table 4.

**Figure 17:**
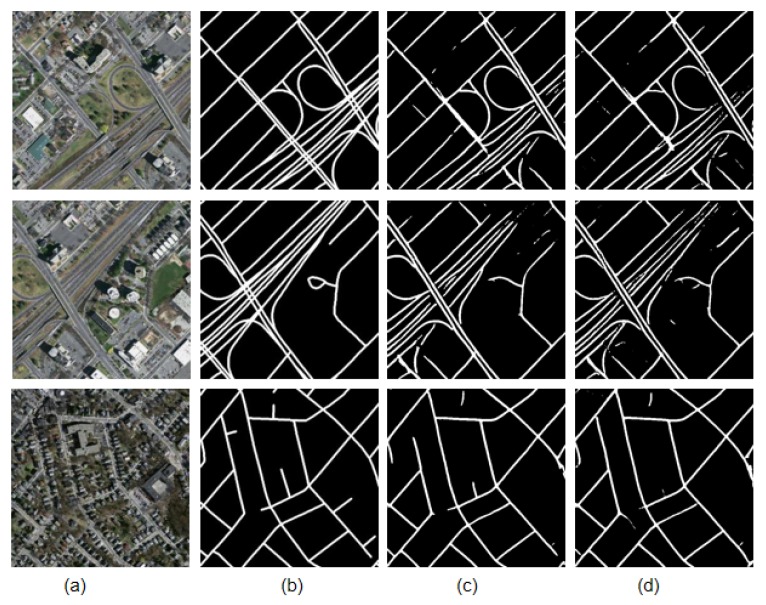
Comparison results of complex scene experiment. (**a**) Input image; (**b**) label image; (**c**) D-LinkNet_50; and (**d**) D-LinkNet_101.

**Figure 18:**
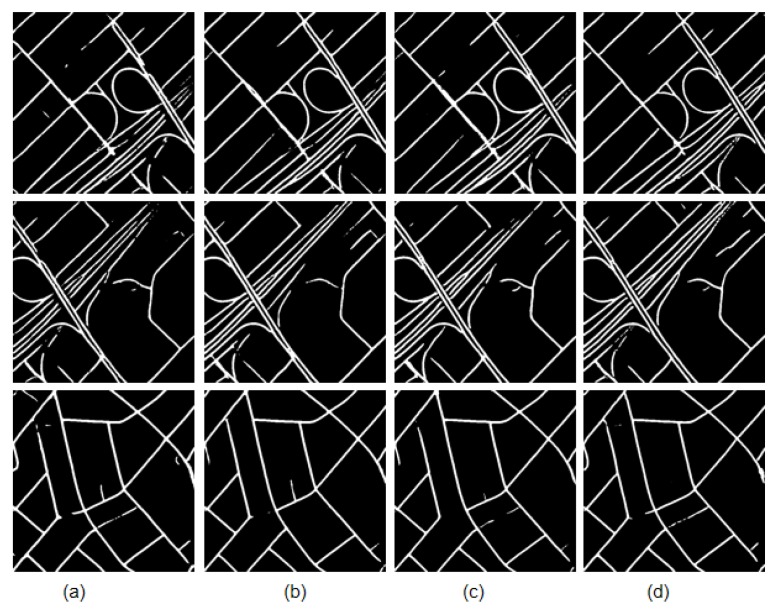
Comparison results of complex scene experiment. (**a**) D-LinkNetPlus_50; (**b**) D-LinkNetPlus_101; (**c**) B-D-LinkNetPlus_50; and (**d**) B-D-LinkNetPlus_101.

The comparison of experimental results of these six models are shown in Figure 17 and Figure 18, respectively.

According to the precision results calculated by IoU with the validation set, for D-LinkNetPlus and B-D-LinkNetPlus, the IoU of the road detection results are better than that of D-LinkNet. Meanwhile, the size of B-D-LinkNetPlus is also better than D-LinkNet. The extracted result of D-LinkNetPlus is similar to that of D-LinkNet because a certain gap existed in the images in comparison with the label. But D-LinkNetPlus greatly reduces model parameters and size; it also shortens the model training time. With a focus on the extracted results from the Massachusetts Roads Dataset, we can see that B-D-LinkNetPlus is the most effective among the three nets. In terms of the B-D-LinkNetPlus_101, it yielded better results in comparison with the other network models. The results are also consistent with the previous analysis on the results of different scenes.

In addition, to further analyze the results of the model B-D-LinkNetPlus_101, different rotating images were tested and analyzed. We took an image and rotated it counterclockwise 30°, 45°, and 60°. Further, we inputted the four images into the B-D-LinkNetPlus_101 for prediction, and then transformed them back to their original orientation. The experimental results show that the IoU of four output results are different. The research on data augmentation for deep learning is interesting and valuable. Accordingly, the sensitivity of rotation should be given more attention in our future work.

## 4. Conclusions

The article presented improved neural networks to extract road information from remote sensing images using a camera sensor equipped with UAV. Because training data play a key role in deep learning method, the sensitivity of the rotation is discussed to add images for training in the article. The D-LinkNet was then introduced for road extraction, and the variant of ResNet was adopted to improve the neural net, known as D-LinkNetplus, which has fewer parameters but offers similar IoU scores. However, the large net size still needs a large amount of calculation; accordingly, the D-block was modified to further develop the improved neural net called B-D-LinkNetPlus. The comparison among the original D-LinkNet_50, D-LinkNet_101, D-LinkNetPlus_50, D-LinkNetPlus_101, B-D-LinkNetPlus_50, and B-D-LinkNetPlus_101 were analyzed. The results were listed to compare the two-evaluator indicator, the model size, and IoU scores. The improved neural net known as B-D-LinkNetPlus_101 was the best choice because of its higher IoU score and smaller net size. Verification was also made using the public Massachusetts Roads Dataset, and the results proved the value of the improved neural net. Further, the research is helpful for extracting information from typical targets using conventional neural nets in remote sensing fields.

## Figures and Tables

**Table 1:** D-LinkNetPlus structure parameters.

Layer Name	Output Size	D-LinkNetPlus_50	D-LinkNetPlus_101
Stem block	128×128	Conv $\left[\begin{array}{c} 3\times 3,k,stride=2\\ 3\times 3,k,stride=1\\ 3\times 3,k,stride=1 \end{array}\right]$	Conv $\left[\begin{array}{c} 3\times 3,k,stride=2\\ 3\times 3,k,stride=1\\ 3\times 3,k,stride=1 \end{array}\right]$
		2×2, avg pool, stride=2	2×2, avg pool, stride=2
Encoder1	128×128	Conv $\left[\begin{array}{c} 1\times 1,64\\ 3\times 3,64\\ 1\times 1,256 \end{array}\right]\times 3$	Conv $\left[\begin{array}{c} 1\times 1,64\\ 3\times 3,64\\ 1\times 1,256 \end{array}\right]\times 3$
Encoder2	64×64	Conv $\left[\begin{array}{c} 1\times 1,128\\ 3\times 3,128\\ 1\times 1,512 \end{array}\right]\times 4$	Conv $\left[\begin{array}{c} 1\times 1,128\\ 3\times 3,128\\ 1\times 1,512 \end{array}\right]\times 4$
Encoder3	32×32	Conv $\left[\begin{array}{c} 1\times 1,256\\ 3\times 3,256\\ 1\times 1,1024 \end{array}\right]\times 6$	Conv $\left[\begin{array}{c} 1\times 1,256\\ 3\times 3,256\\ 1\times 1,1024 \end{array}\right]\times 23$
Encoder4	16×16	Conv $\left[\begin{array}{c} 1\times 1,512\\ 3\times 3,512\\ 1\times 1,2048 \end{array}\right]\times 3$	Conv $\left[\begin{array}{c} 1\times 1,512\\ 3\times 3,512\\ 1\times 1,2048 \end{array}\right]\times 3$
Center	16×16	DBlock	DBlock
Decoder4	32×32	$\left[\begin{array}{c} Conv,1\times 1,512\\ Deconv,3\times 3,512\\ Conv,1\times 1,1024 \end{array}\right]$	$\left[\begin{array}{c} Conv,1\times 1,512\\ Deconv,3\times 3,512\\ Conv,1\times 1,1024 \end{array}\right]$
Decoder3	64×64	$\left[\begin{array}{c} Conv,1\times 1,256\\ Deconv,3\times 3,256\\ Conv,1\times 1,512 \end{array}\right]$	$\left[\begin{array}{c} Conv,1\times 1,256\\ Deconv,3\times 3,256\\ Conv,1\times 1,512 \end{array}\right]$
Decoder2	128×128	$\left[\begin{array}{c} Conv,1\times 1,128\\ Deconv,3\times 3,128\\ Conv,1\times 1,256 \end{array}\right]$	$\left[\begin{array}{c} Conv,1\times 1,128\\ Deconv,3\times 3,128\\ Conv,1\times 1,256 \end{array}\right]$
Decoder1	256×256	$\left[\begin{array}{c} Conv,1\times 1,64\\ Deconv,3\times 3,64\\ Conv,1\times 1,128 \end{array}\right]$	$\left[\begin{array}{c} Conv,1\times 1,64\\ Deconv,3\times 3,64\\ Conv,1\times 1,128 \end{array}\right]$
F1	512×512	Deconv [4×4,32]	Deconv [4×4,32]
Logits	512×512	Conv [3×3,C,stride=1]	Conv [3×3,C,stride=1]

**Table 2:** Comparison results of network size and road precision between D-LinkNet and D-LinkNetPlus.

Network Name	Network Size	IoU
D-LinkNet_50	792 M	51.02%
D-LinkNet_101	0.98 G	52.67%
D-LinkNetPlus_50	686 M	51.85%
D-LinkNetPlus_101	758 M	52.87%

**Table 3:** D-LinkNet and B-D-LinkNetPlus network size and precision comparison results.

Network Name	Network Model Size	IoU
D-LinkNet_50	792 M	51.02%
D-LinkNet_101	0.98 G	52.67%
B-D-LinkNetPlus_50	298 M	52.86%
B-D-LinkNetPlus_101	370 M	52.94%

**Table 4:** Comparison results of road precision between D-LinkNet, D-LinkNetPlus, and B-D-LinkNetPlus.

Network Name	Network Model Size	IoU
D-LinkNet_50	702 M	57.18%
D-LinkNet_101	758 M	57.43%
D-LinkNetPlus_50	582 M	57.58%
D-LinkNetPlus_101	636 M	57.64%
B-D-LinkNetPlus_50	371 M	59.29%
B-D-LinkNetPlus_101	434 M	59.45%
